# OpenVariant: a toolkit to parse and operate multiple input file formats

**DOI:** 10.1093/bioinformatics/btae714

**Published:** 2024-12-02

**Authors:** David Martínez-Millán, Federica Brando, Miguel L. Grau, Mònica Sánchez-Guixé, Carlos López-Elorduy, Iker Reyes-Salazar, Jordi Deu-Pons, Núria López-Bigas, Abel González-Pérez

**Affiliations:** Institute for Research in Biomedicine (IRB Barcelona), The Barcelona Institute of Science and Technology, Barcelona, 08028, Spain; Institute for Research in Biomedicine (IRB Barcelona), The Barcelona Institute of Science and Technology, Barcelona, 08028, Spain; Institute for Research in Biomedicine (IRB Barcelona), The Barcelona Institute of Science and Technology, Barcelona, 08028, Spain; Institute for Research in Biomedicine (IRB Barcelona), The Barcelona Institute of Science and Technology, Barcelona, 08028, Spain; Centro de Investigación Biomédica en Red en Cáncer (CIBERONC), Instituto de Salud Carlos III, Madrid, 28029, Spain; Institute for Research in Biomedicine (IRB Barcelona), The Barcelona Institute of Science and Technology, Barcelona, 08028, Spain; Institute for Research in Biomedicine (IRB Barcelona), The Barcelona Institute of Science and Technology, Barcelona, 08028, Spain; Institute for Research in Biomedicine (IRB Barcelona), The Barcelona Institute of Science and Technology, Barcelona, 08028, Spain; Centro de Investigación Biomédica en Red en Cáncer (CIBERONC), Instituto de Salud Carlos III, Madrid, 28029, Spain; Institute for Research in Biomedicine (IRB Barcelona), The Barcelona Institute of Science and Technology, Barcelona, 08028, Spain; Centro de Investigación Biomédica en Red en Cáncer (CIBERONC), Instituto de Salud Carlos III, Madrid, 28029, Spain; Department of Medicine and Life Sciences, Universitat Pompeu Fabra, Barcelona, 08003, Spain; Institució Catalana de Recerca i Estudis Avançats (ICREA), Barcelona, 08010, Spain; Institute for Research in Biomedicine (IRB Barcelona), The Barcelona Institute of Science and Technology, Barcelona, 08028, Spain; Centro de Investigación Biomédica en Red en Cáncer (CIBERONC), Instituto de Salud Carlos III, Madrid, 28029, Spain; Department of Medicine and Life Sciences, Universitat Pompeu Fabra, Barcelona, 08003, Spain

## Abstract

**Summary:**

Advances in high-throughput DNA sequencing technologies and decreasing costs have fueled the identification of small genetic variants (such as single nucleotide variants and indels) across tumors. Despite efforts to standardize variant formats and vocabularies, many sources of variability persist across databases and computational tools that annotate variants, hindering their integration within cancer genomic analyses. In this context, we present OpenVariant, an easily extendable Python package that facilitates seamless reading, parsing and refinement of diverse input file formats in a customizable structure, all within a single process.

**Availability and implementation:**

OpenVariant is an open-source package available at https://github.com/bbglab/openvariant. Documentation may be found at https://openvariant.readthedocs.io.

## 1 Introduction

The recent advances in sequencing technologies and the associated drop in sequencing prices have fueled the identification of somatic single nucleotide variants (SNVs) and short indels (collectively, mutations) in tumors. This has facilitated numerous studies in cancer genomics that—in the course of less than two decades—have revolutionized our understanding of somatic mutational processes and our knowledge of the genetic roots of cancer (Hudson *et al.* 2010, Weinstein *et al.* 2013, Bailey *et al.* 2018, ICGC/TCGA Pan-Cancer Analysis of Whole Genomes Consortium 2020). These advances, and their application to personalized cancer medicine, rest upon our capability to integrate large numbers of mutations identified across projects (Tamborero *et al.* 2018, Martínez-Jiménez *et al.* 2020, Muiños *et al.* 2021) carried out in different centers, using different sequencing technologies, and variant calling and annotation pipelines (Wang *et al.* 2010, McLaren *et al.* 2016). Despite efforts to homogenize the formats produced by different variant callers (Danecek *et al.* 2011) and the availability of tools and software, such as SAMTools (Li *et al.* 2009), VCFtools (Danecek *et al.* 2011), VEP (McLaren *et al.* 2016), Maftools (Mayakonda *et al.* 2018), and OpenCRAVAT (Pagel *et al.* 2020) to process and annotate variants, differences in the variants produced by various projects persist. These include the usage of different reference genomes, slightly different vocabularies to encode SNVs or indels, the production of different output formats (e.g. VCF or MAF), as well as different variant annotation vocabularies and tools. This variability hinders the task of integrating somatic mutations from different sources, which is key for the success of large cancer genomics analyses. Moreover, currently no tool solves the problem of including metadata relative to mutational datasets obtained from different cohorts of tumors (e.g. dataset name, working directory name, file name), to allow the automated integrated analysis of cohorts sequenced by different projects. The existing systems, such as the ClinGen Allele Registry (Pawliczek et al., 2018) and the GA4GH Variation Representation Specification (VRS) (Wagner et al., 2021), aim to address the limitations of current frameworks and tackle the challenges of large-scale data aggregation. However, these approaches lack versatility and are unable to operate independently of database linkage. Here, we introduce OpenVariant (https://github.com/bbglab/openvariant), a comprehensive Python (Van Rossum and Drake Jr 1995) package that addresses these two problems. On the one hand, OpenVariant encompasses a wide range of functionalities for reading, parsing, and operating multiple variant file formats at once. On the other hand, it manages the annotation of metadata required for automated parallel downstream analysis. Thus, OpenVariant enables the generation of a customized output file that combines all the different input files with a proper annotation file structure. In summary, OpenVariant provides an efficient solution that simplifies and expedites the processes of mutation data cleaning and data management and integration to support the success of cancer genomics analysis.

## 2 Design, implementation, and availability

**Figure 1.**
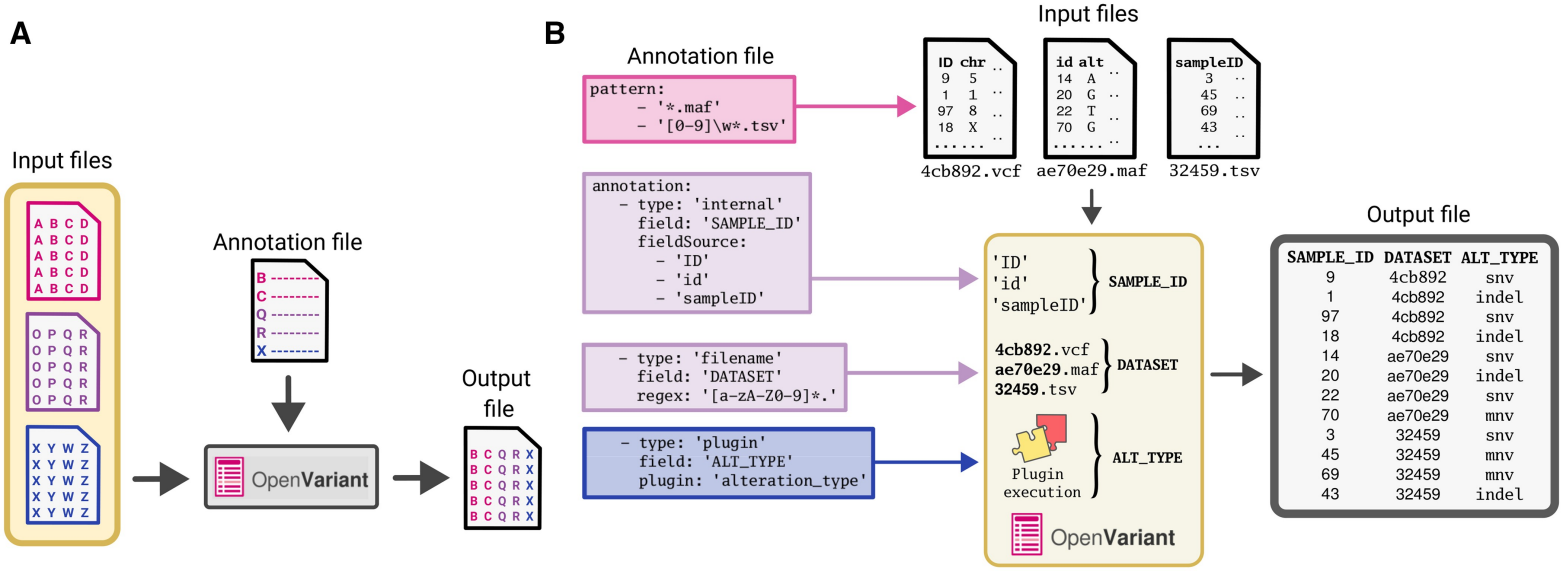
The essential functionality of OpenVariant with the orchestration of all its components. (A) Schematic representation of data workflow and file composition. This diagram shows the interplay between the three main components: input files, annotation file and output file, displaying how the data is processed. (B) Basic parsing functionality of the annotation structure. It illustrates the extraction of three distinct fields from diverse input formats

OpenVariant is a versatile toolkit designed to facilitate the transformation, manipulation, and parsing of mutation data, and cohort metadata with multiple formats. The package can be installed and imported as any other Python package in a script or run through a command-line interface within a shell. The OpenVariant workflow comprises three essential components: input files, annotation files, and output file (one per cohort), as visually illustrated in Fig. 1A. The input files and the annotation files are provided by the user and OpenVariant returns the output file(s). The software extracts a set of rules from the annotation file(s) structure and, then, they are applied to determine how the input files are read, processed and transformed. Upon completion of the entire process, one output file per cohort contains the resulting transformed dataset.

OpenVariant has been developed to ensure efficiency. The package incorporates parallel execution capabilities in some of its functionalities to exploit all available computational resources and expedite processing. The complexity time of the transformation displays a linear relationship with the number of different data conversions done during the parsing process, although the running time may vary based on the complexity of each data transformation.

### 2.1 Annotation structure

The annotation structure serves as a core component which describes how input files are parsed and how the output is represented. The annotation file may describe general properties relevant for parsing and organizing the data, such as patterns in the name of the input files to be included in the analysis, (e.g. different file formats), the format desired for the output, specific columns to be added to the output file, as well as columns of the input files to be omitted in the output file. Additionally, the annotation file specifies all the fields to be parsed and modified by OpenVariant, to manage the conversion of input files to output file(s) (Fig. 1B). The OpenVariant documentation offers an extensive explanation of these properties and their respective data transformation possibilities.

### 2.2 Basic functionalities

OpenVariant encompasses a range of diverse functionalities facilitating data curation and subsequent analyses. The software reads and parses the different input files according to the annotation structure explained above. OpenVariant has been designed to perform four different tasks to aid collating and organizing data for subsequent analysis:

- Find files: given a path, OpenVariant retrieves all input files (with different formats) and the corresponding annotation files that match the pattern parameter.
- Cat: shows the parsed result through the standard output.
- Group-by: retrieves the parsed result grouped based on distinct values of a specific field.
- Count: returns the number of rows from the parsed result that match a specific condition.

These methods can be run as part of a Python script or in a command-line interface. They can be integrated in any pipeline workflow enabling the transformation of the input data as a step within an analysis. Group-by and count tasks have been designed to be executed in parallel. Further details regarding the methods and command-line features may be found in the OpenVariant documentation (https://openvariant.readthedocs.io).

### 2.3 Plugin system

These basic functionalities may be readily extended through plugins implementing specific data transformation tasks. Some plugins already available within the OpenVariant package are described in Supplementary Methods. OpenVariant’s plugin system is inspired by the Ensembl Variant Effect Predictor (VEP; Yourshaw *et al.* 2015).

An OpenVariant plugin is composed of two parts: the Context and the Plugin itself. The Context represents a class that contains several properties of the file being parsed at that moment and the input line that is currently being processed. On the other hand, the Plugin is a class that encompasses the function that is executed to achieve data transformation. OpenVariant simplifies the generation of a plugin template through a simple command, facilitating the customization of data transformation to suit specific user requirements. The software was built focusing on scalability and flexibility, providing the user with the ability to improve the preprocessing steps in tandem with reading and parsing their input data.

## 3 Comparison with existing tools

No currently existing tool possesses the exact same functionalities as OpenVariant. While some tools facilitate the parsing of cancer genomic data with a particular format, to our knowledge, none accepts multiple file formats simultaneously. This distinct feature, combined with the possibility to annotate input datasets using metadata and carry operations on them, makes OpenVariant exceptionally useful and sets it apart from other tools in the field. This unique character of OpenVariant is manifested in Supplementary Table S1 that compares it to other commonly used tools in cancer genomics.

Furthermore, we evaluated the performance of OpenVariant by comparing its execution time to similar Python-based tools. This comparison was conducted using the input files provided by Pedersen (2021). We executed OpenVariant on the VCF example, achieving a median runtime of 26 s in real time (Supplementary Table S2). It is important to note that this runtime may vary if data conversion steps are introduced in the process.

## 4 Usage

To demonstrate the utility of OpenVariant, we integrated it as the first step in the Integrative OncoGenomics (IntOGen) pipeline, aimed at the systematic identification of mutational cancer driver genes across cohorts of tumors deposited in the public domain (Martínez-Jiménez *et al.* 2020). In the context of the latest IntOGen analysis, including 257 898 749 somatic mutations across 33 218 tumor samples, OpenVariant plays an important role in efficiently handling and correctly annotating datasets representing 271 cohorts sequenced by different projects, and stored in different data formats, including CSVs, TSVs, MAFs, and VCFs. The high flexibility of the software allowed it to tailor the parsing process to the specific IntOGen requirements. The integration with the tool enhanced the preprocessing step along with the robustness and adaptability of the pipeline to different format data.

## 5 Conclusion

We present OpenVariant, a scalable and comprehensive Python package designed to facilitate reading, parsing and manipulation of multiple input file formats. The aim of this package is to simplify the curation step and enable researchers to work with diverse formats within a unified workflow. This software package addresses two challenges inherent in high-throughput DNA sequencing analysis: managing multiple variant formats and vocabularies, and enabling the incorporation of metadata associated with the ongoing dataset analysis. The initial outcomes of its performance are highlighted in the IntOGen pipeline. As a real case, it has the capability to efficiently process and convert a significant amount of mutations. We believe that OpenVariant is a novel and indispensable tool that efficiently archives a task valuable in the field. We could not find any comparable solution that performs analogous tasks. The conventional statistical and data analysis packages may achieve this similar goal but their suitability is compromised due to their lack of specialization in the specific task, resulting in their usability being worse. OpenVariant has been developed as an open-source software, designed in an easily extendable way to encourage collaboration in its development and promote the enhancement of its functionalities across diverse use cases. Its extensibility allows users to contribute to its ongoing development and for their own requirements.

## Supplementary Material

btae714_Supplementary_Data

## Data Availability

OpenVariant is open-source and is freely available for public use under BSD-3 Clause license (OSI; Open Source Initiative). This is a permissive license that prohibits the utilization of the copyright holder’s or contributors’ names to endorse derivative products without obtaining prior written consent. OpenVariant may be obtained via the Python Package Index (PyPI; Python Software Foundation 2024).

## References

[btae714-B1] Bailey MH , Tokheim C, Porta-Pardo E et al; MC3 Working Group, Cancer Genome Atlas Research Network. Comprehensive characterization of cancer driver genes and mutations. Cell 2018;173:371–85.e18. 29625053 10.1016/j.cell.2018.02.060 PMC6029450

[btae714-B2] Danecek P , Auton A, Abecasis G et al; 1000 Genomes Project Analysis Group. The variant call format and VCFtools. Bioinformatics 2011;27:2156–8. 21653522 10.1093/bioinformatics/btr330 PMC3137218

[btae714-B3] Hudson TJ , Anderson W, Artez A et al; International Cancer Genome Consortium. International network of cancer genome projects. Nature 2010;464:993–8. 20393554 10.1038/nature08987 PMC2902243

[btae714-B4] ICGC/TCGA Pan-Cancer Analysis of Whole Genomes Consortium. Pan-cancer analysis of whole genomes. Nature 2020;578:82–93. 32025007 10.1038/s41586-020-1969-6 PMC7025898

[btae714-B5] Li H , Handsaker B, Wysoker A et al; 1000 Genome Project Data Processing Subgroup. The sequence alignment/map format and SAMtools. Bioinformatics 2009;25:2078–9. 19505943 10.1093/bioinformatics/btp352 PMC2723002

[btae714-B6] Martínez-Jiménez F , Muiños F, Sentís I et al A compendium of mutational cancer driver genes. Nat Rev Cancer 2020;20:555–72. 32778778 10.1038/s41568-020-0290-x

[btae714-B7] Mayakonda A , Lin D-C, Assenov Y et al Maftools: efficient and comprehensive analysis of somatic variants in cancer. Genome Res 2018;28:1747–56. 30341162 10.1101/gr.239244.118 PMC6211645

[btae714-B8] McLaren W , Gil L, Hunt SE et al The ensembl variant effect predictor. Genome Biol 2016;17:122. 27268795 10.1186/s13059-016-0974-4 PMC4893825

[btae714-B9] Muiños F , Martínez-Jiménez F, Pich O et al In silico saturation mutagenesis of cancer genes. Nature 2021;596:428–32. 34321661 10.1038/s41586-021-03771-1

[btae714-B10] Open Source Initiative. The 3-clause bsd license, 1999. https://opensource.org/licenses/BSD-3-Clause

[btae714-B11] Pagel KA , Kim R, Moad K et al Integrated informatics analysis of cancer-related variants. JCO Clin Cancer Inform 2020;4:310–7. 32228266 10.1200/CCI.19.00132 PMC7113103

[btae714-B4755381] Pawliczek P, , Patel RY, , Ashmore LR et al Clingen allele registry links information about genetic variants. HumanMutation 2018;39:1690–701. 10.1002/humu.23637 PMC6519371 30311374

[btae714-B12] Pedersen B. vcf-bench. 2021. https://github.com/brentp/vcf-bench

[btae714-B13] Python Software Foundation. Python package index. 2024. https://pypi.org/

[btae714-B14] Tamborero D , Rubio-Perez C, Deu-Pons J et al Cancer genome interpreter annotates the biological and clinical relevance of tumor alterations. Genome Med 2018;10:25. 29592813 10.1186/s13073-018-0531-8 PMC5875005

[btae714-B15] Van Rossum G , Drake FL Jr. Python Reference Manual. Centrum voor Wiskunde en Informatica Amsterdam, Amsterdam, Netherlands: University of Amsterdam, 1995.

[btae714-B7424117] Wagner AH, , Babb L, , Alterovitz G et al The GA4GH variation representation specification: A computational framework for variation representation and federated identification. Cell Genomics 2021;1:100027. 35311178 10.1016/j.xgen.2021.100027 PMC8929418

[btae714-B16] Wang K , Li M, Hakonarson H. ANNOVAR: functional annotation of genetic variants from high-throughput sequencing data. Nucleic Acids Res 2010;38:e164. 20601685 10.1093/nar/gkq603 PMC2938201

[btae714-B17] Weinstein JN , Collisson EA, Mills GB et al; Cancer Genome Atlas Research Network. The cancer genome atlas Pan-Cancer analysis project. Nat. Genet 2013;45:1113–20. 24071849 10.1038/ng.2764 PMC3919969

[btae714-B18] Yourshaw M , Taylor SP, Rao AR et al Rich annotation of DNA sequencing variants by leveraging the ensembl variant effect predictor with plugins. Brief Bioinform 2015;16:255–64. 24626529 10.1093/bib/bbu008 PMC6283364

