# Supplementary material for: OpenVariant: a toolkit to parse and operate multiple input file formats
Source: Bioinformatics. 2024 Dec 2;40(12):btae714. doi: 10.1093/bioinformatics/btae714 (PMC11634536; doi:10.1093/bioinformatics/btae714)
Supplement: btae714_Supplementary_Data [file btae714_supplementary_data.zip › openvariant-supplementary[AU].pdf]

Supplementary Data

OpenVariant: a toolkit to parse and operate multiple input file formats

David Martínez-Millán [1], Federica Brando [1], Miguel L. Grau [1], Mònica Sánchez-Guixé [1,2], Carlos López-Elorduy [1], Iker Reyes-Salazar,[1] Jordi Deu-Pons [1,2], Núria López-Bigas [1,2,3,4] and Abel González-Pérez [1,2,3]

[1]Institute for Research in Biomedicine (IRB Barcelona), The Barcelona Institute of Science and Technology, Barcelona, 08028, Spain, [2]Centro de Investigación Biomédica en Red en Cáncer (CIBERONC), Instituto de Salud Carlos III, Madrid, 28029, Spain, [3]Department of Experimental and Health Sciences, Universitat Pompeu Fabra, Barcelona, 08003, Spain and [4]Institució Catalana de Recerca i Estudis Avançats (ICREA), Barcelona, 08010, Spain

1. Supplementary Methods

1.1. Classification of alteration type

This plugin acquires the mutation type from each parsed mutation that is read in the process. It defines whether the mutation is a Multi-nucleotide Variant (MNV), characterized by two or more variants existing on the same haplotype in an individual, a Single-nucleotide Variant (SNV), denoting a variant of a single nucleotide that occurs at a specific genomic position, or if it is a deletion or an insertion (INDEL). The plugin extracts the alteration, the reference and the position value from the mutation, to determine the number of variants and then classify it.

1.2. Alternate allele frequency

Alternate allele frequency is a field provided in Variant Call Format (VCF) files (1000 Genome Project Data Processing Subgroup (2022)), which refers to the proportion of variant alleles within a genomic locus, and provides insights into tumor clonality in somatic genomic testing (Boscolo Bielo et al. (2023)). It is one of the most used values for filtering variants in a somatic mutation workflow. The present plugin extracts this value using a regular expression (van Leeuwen (1991)).

1.3. LiftOver operation

The LiftOver plugin is a functionality that supports the conversion of genomic coordinates, ensuring compatibility across assemblies in the dataset. Powered by the pyliftover library (Tretyakov (2013)), this plugin currently supports conversions between hg19 and hg38 assemblies. Users can specify the source and target genome assemblies in the annotation, allowing OpenVariant to perform LiftOver along with data parsing. The plugin extracts the position value from the input and performs the conversion from hg19 to hg38 or vice versa, given the information the user specified in the annotation file.

2. Supplementary Tables

Table S1: Comparing OpenVariant to several analogous tools. It provides a comprehensive understanding of the multiple possibilities that OpenVariant offers and similarities and differences across various features in contrast to other tools.

Tool name	Reference	Programming language	Input formats	Output formats	Mix multiple file formats	Annotation structure	Metadata annotation	Parsing	Filtering	Grouping	Parallel computing	Compressing
OpenVariant	-	Python	CSV, TSV, MAF, VCF	TSV, CSV	Yes	Yes	Yes	Yes	Yes	Yes	Yes	No
vembrane	Hartmann et al. (2022)	Python	VCF, BCF	TSV, VCF, BCF	No	Yes	No	Yes	Yes	No	No	No
vcflib	Garrison et al. (2022)	C++/Python	VCF	VCF	No	No	No	Yes	Yes	No	Yes	No
slivar	Pedersen et al. (2021)	Javascript	VCF	TSV	No	No	No	Yes	Yes	Yes	No	No
bio-vcf	Garrison et al. (2021)	Ruby	VCF	TSV, JSON, RDF, VCF	No	Yes	Yes	Yes	Yes	No	Yes	No
SAMTools	Li et al. (2009)	C	VCF, IMPUTE2, GVCF, HAP/LEGEND, TSV, BAM, CRAM	VCF, GEN/SAMPLE, FASTA, FASTAQ, BAM	No	No	No	Yes	Yes	Yes	Yes	Yes
HTSlib	Bonfield et al. (2021)	C	SAM, BAM, CRAM, VCF, BCF	SAM, BAM, CRAM, VCF, BCF	No	No	No	Yes	No	No	Yes	Yes
vcf2maf	Kandoth et al. (2013)	Perl	VCF, MAF	MAF	No	No	No	Yes	Yes	No	No	No
maftools	Mayakonda et al. (2018)	R	ANNOVAR, ICGC Simple Somatic Mutation, MAF, VCF	MAF, BCF	No	No	No	Yes	Yes	No	No	No
Vcfanno	Pedersen et al. (2016)	Go	VCF, BED, BAM, GFF, GTF	VCF	No	Yes	No	Yes	No	No	Yes	No
VCF-kit	Cook (2014)	Go	VCF	TSV, VCF	No	No	No	Yes	Yes	No	No	No
jvarkit	Lindenbaum (2015)	Java	BAM, GTEx, GTF, GFF3, IBD, CLUSTAW, FASTA, SAM	VCF, raster graphics, SQLITE, SVG, BedGraph, Wiggle, XML, QTL, BED, FASTA, GFF3, HTML, JSON, TSV	No	No	No	Yes	Yes	Yes	No	Yes
pysam	Heger et al. (2023)	Python	SAM, BAM, VCF, BCF, BED, GFF, GTF, FASTA, FASTQ	BAM, CRAM, SAM	No	No	No	No	No	No	Yes	Yes
VCFtools	Danecek et al. (2011)	Perl/C++	VCF, BCF	VCF, IMPUTE, LDhat/LDhelmet, BEAGLE.GL, BEAGLE.PL, PED, MAP	No	No	No	Yes	Yes	No	No	Yes
PyVCF	Dougherty et al. (2011)	Python	VCF	VCF	No	No	Yes, metadata fields from header	Yes	Yes	No	No	No
cyvcf2	Pedersen and Quinlan (2017)	Python	VCF, BCF	-	No	No	No	No	Yes	No	Yes	No
VEP	McLaren et al. (2016)	Perl	VCF	TSV	No	No	No	Yes	Yes	No	No	No
annonex2embl	Gruenstaeudl (2020)	Python	NEX, CSV	Multi-record EMBL flatfile	No	No	No	Yes	No	No	No	Yes
vcf-js	Diesh et al. (2018)	TypeScript	VCF	-	No	No	No	No	No	No	No	No
OpenCRAVAT	Pagel et al. (2020)	Python	TSV, VCF, Ancestry.com, 23andMe, FamilyTreeDNA	Text format, Excel, TSV, CSV, VCF	No	Yes	No	Yes	Yes	Yes	No	No
ClinGen Allele Registry	Pawliczek et al. (2018)	C++	VCF, HGVS expressions	JSON-LD	No	No	No	Yes	Yes	No	No	No
GA4GH Variation Representation Specification (VRS)	Wagner et al. (2021)	Python	Database	VCF, HGVS, dbSNP	No	Yes	No	Yes	Yes	No	No	No

Table S2: Comparing execution time of individuals runs in different Python-based tools with a VCF file provided by Pedersen (2021).

**Tool name**	**Execution time**
OpenVariant	26s
pysam	26s
cyvcf2	40s
pyvcf	14m

References

1000 Genome Project Data Processing Subgroup. *The Variant Call Format (VCF) Version 4.2 Specification.* Genome Research Limited, 2022. URL `https://samtools.github.io/hts-specs/VCFv4.2.pdf`.

J. K. Bonfield, J. Marshall, P. Danecek, H. Li, V. Ohan, A. Whitwham, T. Keane, and R. M. Davies. HTSlib: C library for reading/writing high-throughput sequencing data. *Gigascience*, 10(2), Feb. 2021.

L. Boscolo Bielo, D. Trapani, M. Repetto, E. Crimini, C. Valenza, C. Belli, C. Criscitiello, A. Marra, V. Subbiah, and G. Curigliano. Variant of allele frequency: a decision-making tool in precision oncology? *Trends Cancer*, Sept. 2023.

D. E. Cook. Vcf-kit. `https://github.com/AndersenLab/VCF-kit`, 2014.

P. Danecek, A. Auton, G. Abecasis, C. A. Albers, E. Banks, M. A. DePristo, R. E. Handsaker, G. Lunter, G. T. Marth, S. T. Sherry, G. McVean, R. Durbin, and 1000 Genomes Project Analysis Group. The variant call format and VCFtools. *Bioinformatics*, 27(15):2156–2158, Aug. 2011.

C. Diesh, G. Stevens, R. Buels, B. Powell, and J. Blom. vcf-js. `https://github.com/GMOD/vcf-js`, 2018.

J. Dougherty, J. Casbon, A. Quinlan, B. Pedersen, M. Vermaat, I. Roberts, and M. Martin. Pyvcf - a variant call format parser for python. `https://github.com/jamescasbon/PyVCF`, 2011.

E. Garrison, Z. N. Kronenberg, E. T. Dawson, B. S. Pedersen, and P. Prins. Vcflib and tools for processing the VCF variant call format. May 2021.

E. Garrison, Z. N. Kronenberg, E. T. Dawson, B. S. Pedersen, and P. Prins. A spectrum of free software tools for processing the vcf variant call format: vcflib, bio-vcf, cyvcf2, hts-nim and slivar. *PLOS Computational Biology*, 18(5):e1009123, May 2022. ISSN 1553-7358. doi: 10.1371/journal.pcbi.1009123. URL `http://dx.doi.org/10.1371/journal.pcbi.1009123`.

M. Gruenstaeudl. annonex2embl: automatic preparation of annotated DNA sequences for bulk submissions to ENA. *Bioinformatics*, 36(12): 3841–3848, June 2020.

T. Hartmann, C. Schröder, E. Kuthe, D. Lähnemann, and J. Köster. Insane in the vembrane: filtering and transforming VCF/BCF files. *Bioinformatics*, 39(1):btac810, 12 2022. ISSN 1367-4811. doi: 10.1093/bioinformatics/btac810. URL `https://doi.org/10.1093/bioinformatics/btac810`.

A. Heger, J. Marshall, and K. Jacobs. pysam: htslib interface for python. `https://github.com/pysam-developers/pysam`, 2023.

C. Kandoth, J. Gao, A. Hoyle, Q. Wang, M. Mattioni, Zuojian-Tang, A. Penson, I. de Bruijn, A. Struck, G. Hogue, Y. Boursin, M. Nakhoul, R. Sheridan, H. Z. Sebastian Lange, and S. Chavan. vcf2maf. `https://github.com/mskcc/vcf2maf`, 2013.

H. Li, B. Handsaker, A. Wysoker, T. Fennell, J. Ruan, N. Homer, G. Marth, G. Abecasis, R. Durbin, and 1000 Genome Project Data Processing Subgroup. The sequence Alignment/Map format and SAMtools. *Bioinformatics*, 25(16):2078–2079, Aug. 2009.

P. Lindenbaum. JVarkit: java-based utilities for bioinformatics, May 2015.

A. Mayakonda, D.-C. Lin, Y. Assenov, C. Plass, and H. P. Koeffler. Maftools: efficient and comprehensive analysis of somatic variants in cancer. *Genome Res.*, 28(11):1747–1756, Nov. 2018.

W. McLaren, L. Gil, S. E. Hunt, H. S. Riat, G. R. S. Ritchie, A. Thormann, P. Flicek, and F. Cunningham. The ensembl variant effect predictor. *Genome Biol.*, 17(1), Dec. 2016.

K. A. Pagel, R. Kim, K. Moad, B. Busby, L. Zheng, C. Tokheim, M. Ryan, and R. Karchin. Integrated informatics analysis of cancer-related variants. *JCO Clin. Cancer Inform.*, 4(4):310–317, Mar. 2020.

P. Pawliczek, R. Y. Patel, L. R. Ashmore, A. R. Jackson, C. Bizon, T. Nelson, B. Powell, R. R. Freimuth, N. Strande, N. Shah, S. Paithankar, M. W. Wright, S. Dwight, J. Zhen, M. Landrum, P. McGarvey, L. Babb, S. E. Plon, and A. Milosavljevic. Clingen allele registry links information about genetic variants. *Human Mutation*, 39(11):1690–1701, Oct. 2018. ISSN 1098-1004.

B. Pedersen. vcf-bench. `https://github.com/brentp/vcf-bench`, 2021.

B. S. Pedersen and A. R. Quinlan. cyvcf2: fast, flexible variant analysis with python. *Bioinformatics*, 33(12):1867–1869, June 2017.

B. S. Pedersen, R. M. Layer, and A. R. Quinlan. Vcfanno: fast, flexible annotation of genetic variants. *Genome Biol.*, 17(1), Dec. 2016.

B. S. Pedersen, J. M. Brown, H. Dashnow, A. D. Wallace, M. Velinder, M. Tristani-Firouzi, J. D. Schiffman, T. Tvrdik, R. Mao, D. H. Best, P. Bayrak-Toydemir, and A. R. Quinlan. Effective variant filtering and expected candidate variant yield in studies of rare human disease. *NPJ Genom. Med.*, 6(1):60, July 2021.

K. Tretyakov. pyliftover. `https://github.com/konstantint/pyliftover`, 2013.

J. van Leeuwen, editor. *Handbook of theoretical computer science (vol. A): algorithms and complexity.* MIT Press, Cambridge, MA, USA, 1991. ISBN 0444880712.

A. H. Wagner, L. Babb, G. Alterovitz, M. Baudis, M. Brush, D. L. Cameron, M. Cline, M. Griffith, O. L. Griffith, S. E. Hunt, D. Kreda, J. M. Lee, S. Li, J. Lopez, E. Moyer, T. Nelson, R. Y. Patel, K. Riehle, P. N. Robinson, S. Rynearson, H. Schuilenburg, K. Tsukanov, B. Walsh, M. Konopko, H. L. Rehm, A. D. Yates, R. R. Freimuth, and R. K. Hart. The ga4gh variation representation specification: A computational framework for variation representation and federated identification. *Cell Genomics*, 1(2):100027, Nov. 2021. ISSN 2666-979X.
